# Competence, commitment and opportunity: an exploration of faculty views and perceptions on community- based education

**DOI:** 10.1186/1472-6920-13-167

**Published:** 2013-12-13

**Authors:** Zahra Ladhani, Fred J Stevens, Albert J Scherpbier

**Affiliations:** 1 Shifa College of Nursing, Islamabad, Pakistan; 2 Department of Educational Development & Research, Maastricht University, Maastricht, the Netherlands; 3 Faculty of Health, Medicine and Life Sciences, Maastricht University, Maastricht, the Netherlands

## Abstract

**Background:**

Community-Based Education (CBE) is an instructional approach designed and carried out in a community context and environment in which not only students, but also faculty and Health Professionals’ Education (HPE) institutions must be actively engaged throughout the educational experience. Despite the growing evidence of CBE being an effective approach for contemporary HPE, doubts about its successful implementation still exist. This study has explored HPE structure, policies and curriculum from the point of view of faculty members to gain understanding about the prevailing practices and to propose recommendations that nurtures and promotes CBE.

**Method:**

A purposive sample was drawn from three major cities of Pakistan- Karachi, Rawalpindi and Islamabad. Out of twelve HPE institutions present in these cities we selected six, which provided a sound representation of medical and nursing colleges around the country. At each institution we had two Focus Group Discussions; in addition we interviewed registrars of medical and nursing councils and two CBE experts.

**Results:**

The factors effecting implementation of CBE as perceived by study participants are categorized as: preparation of faculty members; institutional commitment and enthusiasm; curricular priorities and external milieu. Within each theme, participants recurrently described structural and curricular deficiencies, and lack of commitment and appreciation for community based teaching, service and research permeating at all levels: regulatory bodies, institutional heads and faculty members.

**Conclusions:**

The factors highlighted by our study and many others suggest that CBE could not perpetuate effectively within HPE. To enhance the effectiveness of CBE approach in a way that mutually benefits local communities as well as HPE institutions and health professionals, it is important that reforms in HPE must be strategized in a holistic fashion i.e. restructuring and aligning its polices, curriculum and research priorities.

## Background

Community-Based Education (CBE) is an instructional approach designed and carried out in a community context, outside the teaching hospital [1,2]. It consists of learning activities that must utilize the community extensively as a learning environment in which not only students, but also faculty members are actively involved throughout the educational experience [3]. Over time, a variety of CBE models have emerged around the globe, however, it remains questionable whether CBE is truly integrated within HPE curricula [4-6].

Community-based encounters usually accounts for a very small proportion of a health professional student’s time compared to hospital-based experiences in the clinical years [6-8]; moreover these are not made relevant to the requirements of practice. Umer et al. [9] found that “students have no community experience and at best gain only superficial knowledge from textbooks; although a large number of students had visited primary health care facilities, they seldom interacted with rural communities”. Furthermore, there is growing evidence that there aren’t enough teaching methods that are purposefully designed to support CBE or community service [7-9]. Evaluating a CBE programme, Kristina et al. identified time allocation as another structural issue, reporting that “curricula are earmarked for CBE, but in practice only about half of that time is used for learning in the community, the rest is taken for lectures and activities in facilities remote from the community [6,7]. Thus, despite international academic organisations recognising the importance of community and social orientation of curricula, the conventional curriculum is persisting as the predominant model in HPE [10,11]. The recent report in the Lancet by the commission on Education of Health professionals for the 21st century concludes that HPE is not able to address health care challenges, largely because of fragmented, outdated, and static curricula producing ill-equipped graduates [11].

A previous study on competencies of health professionals explored the tasks and functions of physicians and nurses working in community settings of Pakistan [12]. It was found that curative care was the most frequently performed task even at the primary health care setup. Preventive or promotive health care, screening or other public health activities were not regularly practiced mainly because health professionals were not prepared to take up the roles required at community settings. The same study also informed us that most often the health providers did not receive adequate training or weren’t appraised on the core requirements for community based care during the undergraduate program [12]. Henceforth, for our present study we explored HPE institutional structure, policies and curriculum from the point of view of faculty members. Our intent is to gain understanding about the prevailing practices and to propose a model for health professional education that nurtures and promotes CBE. Documentation of this information as well as potential solutions to the identified challenges in running CBE programs might not only improve the training of health professionals but also assist in the long-term goal of improving the recruitment, deployment or retention of health professionals in community settings.

## Methods

### Sample

A purposive sample was drawn from public, private and armed forces institutions having both-medical and nursing-undergraduate programs, recognized by the regulatory bodies, and the ones located in the three major cities of Pakistan- Karachi (nine), Rawalpindi (one) and Islamabad (two) [13,14]. Out of twelve HPE institutions from the three cities we selected six, providing a sound representation of medical and nursing colleges around the country. At each institution we had two groups of faculty members (5 to 7 members each) for the Focus Group Discussions (FGDs). Additionally, we interviewed registrars of regulatory bodies and two community experts: a senior medical educator involved in initiating CBE in Pakistan and the other was amongst the first few nurses to work as a community health nurse in the country. Approval of the study protocol was obtained from the research ethics committee of Shifa Colleges for Nursing and Medicine, Islamabad, Pakistan.

### Data collection and analysis

Verbal consent from each participant was obtained before beginning the FGDs and interviews. Discussion was generated around areas like: their training and preparation for CBE; departmental research agenda; promotion criteria of the institute and whether community service and or research were inclusive; resource allocation for their and student’s practice in community settings; facilitation and support by their institutions and regulatory bodies. Questions concerning curriculum and teaching included: teaching hours and content; satisfaction with the course and its outcome; their understanding of CB competencies and any specific competencies they consider including in the curriculum. In-depth interviews had similar vein of questions albeit from the point of view of regulation; planning challenges and recommendations for the CBE programme at national level.

FGDs and interviews were conducted by the principal researcher, who took notes and audio recorded the interviews. Since all interviews were conducted in the English language there were no translation issues. The notes were typed up immediately after the interview and, where practically possible, shared with the participants to determine its accuracy. Once notes were approved by all the participants, these were imported into qualitative research software - Nvivo 9, which was used to organise and analyse the data. After listening to and reviewing the recordings, the principal researcher prepared detailed summaries and drafted a list of initial inductive codes, discussed it with the other two authors for refinement and revision. Using the revised codes, the data was re-read, re-coded and categorised to generate themes.

## Results

### Participant characteristics

A total of 65 faculty members, 28 nursing and 37 from medical colleges participated in a total of twelve FGDs. The participants were mainly female i.e. over 90% who were at different stages in their careers (early career 55%, senior faculty 35%, and head of department 10%). Of the faculty from medical colleges, 65% worked full time in the community health/medicine department and the others were co-teaching family medicine. The two (private) nursing colleges had a group of faculty members dedicated full-time to the community health nursing course whereas the faculty of the other three colleges taught multiple courses.

### Perceptions and practices

The factors effecting implementation of CBE as perceived by the faculty members and senior educators could be described under following categories: preparation of faculty members; institutional commitment and enthusiasm; curricular priorities and external milieu.

### Preparation of faculty members

Receiving formal training to be an educator was found to be un-common and preparation for planning and implementing CBE a rarity. Of the medical faculty members, 94% had received their training on a traditional curriculum without much exposure to community settings as an undergraduate student, while 65% of faculty from nursing colleges had similar undergraduate experiences as their medical colleagues. From the total participants, 45% had some sort of formal training a certificate course or diploma or graduate studies in Public (or community Health) while the remaining 50% had training in other speciality areas such as internal or family medicine; Ob-gyn and dermatology and 5% had their basic medical/nursing degree only. Institutional organized workshops and continue education sessions were reported as most frequent means of updating the content knowledge for faculty members. Only a few participants identified community focused teaching, service or research as their expertise or part of current practice.

### Institutional commitment and enthusiasm

Interviews with community experts and registrars of regulatory bodies indicated that academic institutions were not committed to carry forward the CBE agenda: “*HPE institutions are required to adopt one BHU* (*Basic Health Unit*) *to mutually benefit the community and the students*; *however except one or two institutions this requirement was not followed in the country*.” Another illustration was: “*Even though in early 80s a large amount of funding was spent on training for teaching community health nursing*, *the teachers were not put to any use. Once they* (*teachers*) *returned to their institutions*, *they were assigned to teach different subjects*.” At the institutional level, “*issues are also related to resources such as the unavailability of vehicle*; *when there is a vehicle there is no money for petrol*, *shortage of trained teachers in schools and for health facilities*” (Lack of) resources in the form of employing trained personnel and investing in training of faculty members, financing community service and research activities was identified as a major problem in all types of - public, private, and armed forces- institutions. In addition, institution’s promotion policies heavily favoured clinical research and teaching. There were “g*aps*” between expectations and reward systems, “*college expects* (us) *to spend time for community service and teaching but those who do*, *are not recognized as those* (faculty) *in clinical research or teaching in hospital*”, *this* (attitude) *could only be changed when* (national) *leadership would make it* (community service and research) *part of promotion criteria*”. Few participants also identified resistance from administrator and/or senior faculty members to adopt newer approaches of teaching, a community expert stated: “*CBE and PBL demand and expect that departments are dissolved and the curriculum is integrated within a system*, *but the problem is that with this change the power of departments diminishes*, *and therefore there is a lot of resistance*”. Generalized apathy was found among the participants; most believed that change was impossible unless the curriculum was revolutionised by the regulatory bodies: “*these* (curricular and time division) *changes could only be mandated through regulatory bodies*, *and unless they won*’*t take such steps*, *there will be no change*”. Lack of commitment, appreciation of and resistance to CBE appeared to permeate at all levels - regulatory bodies, institutional heads and faculty members.

### Curricular priorities

The academic institutions generally follow the curriculum prescribed by the regulatory bodies which around 85-90% has clinical and tertiary care focus. Community training was mostly considered as imparting knowledge in classroom. Furthermore, a prevailing understanding was community visits could be substituted by consulting patients in out-patient clinic which draws population from all surrounding communities and “*students get ample opportunities to interact with patients from poor neighbourhoods*”. Clinical training in the community consisted of “field visits” i.e. a number of day-trips to places like water filter plant, a factory, incineration plant and a primary or secondary level health care facility, with students being expected to write about their experiences in a “*visit book*” afterwards. The participants who were cognizant about adding variety of learning sites to enhance students’ experiential and contextual learning and its potential benefits to the community, indicated that the one-day trips had little or no relevance to the content taught in the classroom and suggested that the community training should expose students to “*real life conditions*”, considering that most (medical) students are from upper or middle income families and usually have no idea about the living conditions of the mainstream population. However, they voiced that the factors outside their control constraints them to do so, such as large class size; lack of resources and safety concerns in the city. They added, that even though it was important to “*show*” students the real setting, it was not always possible to do so, “*we have resources for 100 students*, *but our college enrols 300 students annually*; *there is no way to plan community based training for such big group. Considering the resources and incentives we get*, *we are doing our best*”. Thus leaving CBE a “*subject*” to be taught in classrooms where it is “*important is to memorize the content to pass exams*”.

### External milieu

The factors and situations outside the academic walls had direct bearing on CBE, including availability of field sites, transport to and within the community, accommodation facilities and safety conditions. Dysfunctional health systems, lack of supervisors and role models at the health facility level were also highlighted by all participants as important factors in determining CBE. On the other hand, involvement of health department, community or other stakeholder was not found to be part of curriculum planning or selecting field sites.

## Discussion

In Pakistan, CBE has existed for over 25 years and accredited medical and nursing schools are required to include some form of CBE in their curricula [15]. However, our findings demonstrate that there are a number of challenges that must to be addressed to truly integrate CBE as an educational approach within HPE. Firstly, community based teaching, service, and research not being a priority of HPE institutions reciprocates lack of interest, attention and poor reward structures for CBE; a number of other studies [5-10] have underscored this finding, for example Joyce and Joanne found that community service commitments were not valued to the same extent as teaching and research, with one of their study participants describing community service as “the stepchild in the tripartite university mission” [16]. Similarly, Bloom reported that a large amount of funding in American medical schools was directed to academic research and specialty medicine and at the same time there was less funding for community teaching and service. Report from Diane and colleagues [17] reiterated that “(unidentified) faculty roles and (poor) rewards policies can be barriers to significant and sustained faculty involvement in communities”.

In relation to the curriculum, community based encounters were considered inadequate and having little relevance to the content that was taught. By and large learning opportunities within community settings were underutilised even though faculty acknowledged that community rotations increased students’ understanding of population problems and provided context for their learning. Previous studies also showed that in general CB teaching is undervalued, research is overvalued, and community service is the least valued of activities [10,16-18].

Moreover, the role of faculty is critical to making CBE work, and our study and many others testify to the fact that this continues to be a challenge, despite considerable investment in faculty development [16,18]. Of the participants in our study more than half were not formally trained for CBE, moreover a large number of participants did not appear to have internalised the real meaning of CBE, with many concepts and competencies unclear. Lubna et al., reported similar results that “unfortunately due to lack of commitment from administration, communication amongst stakeholders and faculty buy in CBE could not be effectively implemented in Pakistan” [15].

### Limitations

This paper has explored perceptions of faculty members to discern the practices and structures of HPE institutions concerning CBE rather than using any objective measure. The intent of the study was to explore CBE implementation or its lack thereof through perspective lens of one of the critical element of HPE i.e. faculty members because it provided an in-depth understanding of the subject under study within the contexts that are dynamic and social in their foundation and structure which are difficult to capture otherwise.

### Recommendations

The perspectives and practices reported by our study participants brought to surface several opportunities where concentrated input could help built the momentum for CBE. First, properly trained and enthusiastic faculty members have huge potential in taking forward the agenda of CBE. It must be recognized that with the advent of various approaches in HPE including CBE, the traditional way to teaching whereby the clinicians and practitioners with no formal training as educators have functioned as faculty members and teach students in much the same way as they were taught is no longer working. In addition, the one-time content based trainings for faculty members are also not showing any sustainable results, hence there is a need for such programs which are geared toward holistic faculty development. More recently, extensive faculty development are being proposed that emphasize attitudes along with knowledge and skills, extend its reach from the individual learner to the entire system, and encompass the whole spectrum of teaching and learning. This broad understanding of faculty development is particularly pertinent to CBE [19].

Secondly, for a strategic and sustainable change, the reforms within HPE institutes must be conceived and delivered in a well balanced manner. The reorganization must aim at improving, stabilizing and aligning all its elements- institutional structure, policies, curriculum, teaching methods, research priorities, faculty and leadership- by ensuring that each of these elements receive a balanced share of recognition and resources; by safeguarding the interest and importance of all levels of care-primary, secondary and tertiary- and by building a culture of community service and civic engagement among faculty and students alike. Unless all elements are well aligned and strengthened together, the peace-meal approach will unlikely change the situation.

Finally, acceptance of and working towards HPE or CBE matter cannot be delivered in a vacuum, it is affected by the society and by its prevalent systems. Presently, HPE is working in its own silo; curriculum planning and delivery is solely carried out within the walls of institutes, researches are hardly partnered and contributions by its faculty or graduates are limited [10,11,14]. For the transformation in its values, beliefs, practices and policies, one valuable action would be to go beyond the walls of HPE and embrace meaningful partnerships with other stakeholders starting from the ones that directly influence CBE such as community based practitioners, representatives from communities, regulatory bodies and policy makers; thus, engaging with the health system while planning the change for HPE.

## Conclusion

Number of investments have been poured into enhancing CBE in Pakistan and worldwide, however due to factors highlighted by our studies and many others suggest that CBE could not perpetuate well within HPE. The implementation of CBE in its true sense demands that strategic and sustainable change in HPE must be strategized in a holistic fashion i.e. reforming all its elements – institutional structure, polies, curriculum and faculty- and that these must be well balanced in terms of resource allocation and commitment at all levels. Additionally, cognizant to the fact that well prepared faculty members are crucial to the success of any HPE endeavour, it is imperative to invest not only in their training but also in creating mechanisms to harness their interest and enthusiasm to advance the promotion and delivery of CBE.

## Competing interests

We declare that there are no conflicts of interest. We are alone responsible for the content and writing of the paper.

## Authors’ contributions

The article is part of the PhD thesis of Dr. ZL who was chiefly responsible for the conception and design of the study, collected and analyzed the data. Drs. FS and AJS supervised and advised throughout the process and in all the steps including drafting the manuscript. All authors have read and approved the final manuscript.

## Pre-publication history

The pre-publication history for this paper can be accessed here:

http://www.biomedcentral.com/1472-6920/13/167/prepub
